# A Comprehensive Review of Computation-Based Metal-Binding Prediction Approaches at the Residue Level

**DOI:** 10.1155/2022/8965712

**Published:** 2022-03-31

**Authors:** Nan Ye, Feng Zhou, Xingchen Liang, Haiting Chai, Jianwei Fan, Bo Li, Jian Zhang

**Affiliations:** ^1^School of Finance and Economics, Xinyang Agriculture and Forestry University, Xinyang 464000, China; ^2^School of Computer and Information Technology, Xinyang Normal University, Xinyang 464000, China; ^3^College of Medical, Veterinary and Life Sciences, University of Glasgow, Glasgow G12 8QQ, UK; ^4^College of Electronic Science and Engineering, Jilin University, Changchun 130012, China

## Abstract

Clear evidence has shown that metal ions strongly connect and delicately tune the dynamic homeostasis in living bodies. They have been proved to be associated with protein structure, stability, regulation, and function. Even small changes in the concentration of metal ions can shift their effects from natural beneficial functions to harmful. This leads to degenerative diseases, malignant tumors, and cancers. Accurate characterizations and predictions of metalloproteins at the residue level promise informative clues to the investigation of intrinsic mechanisms of protein-metal ion interactions. Compared to biophysical or biochemical wet-lab technologies, computational methods provide open web interfaces of high-resolution databases and high-throughput predictors for efficient investigation of metal-binding residues. This review surveys and details 18 public databases of metal-protein binding. We collect a comprehensive set of 44 computation-based methods and classify them into four categories, namely, learning-, docking-, template-, and meta-based methods. We analyze the benchmark datasets, assessment criteria, feature construction, and algorithms. We also compare several methods on two benchmark testing datasets and include a discussion about currently publicly available predictive tools. Finally, we summarize the challenges and underlying limitations of the current studies and propose several prospective directions concerning the future development of the related databases and methods.

## 1. Introduction

Metal ions are certain atom compounds that usually form cations that have (a) positive electric charge(s). Metal ions play pivotal roles in protein structure, function, regulation, and stability [1, 2]. Common metal ions include zinc (Zn^2+^), calcium (Ca^2+^), magnesium (Mg^2+^), manganese (Mn^2+^), iron (Fe^3+^ or Fe^2+^), copper (Cu^2+^), cobalt (Co^2+^), sodium (Na^+^), potassium (K^+^), and nickel (Ni^2+^) ions. Recent estimates have shown that approximately 30%-40% of proteins require one or several metal cofactors to together express biological function [3]. The proportion varies in different types of organisms or tissues. For instance, K^+^ is mostly found inside the cell, while Na^+^ is abundant outside of the cell [4]. Mn^2+^ is found accumulated in leafy green plants [5]. In the human body, Ca^2+^ accounts for approximately 1.5% of total body weight. The bulk of Ca^2+^ is aggregated in bones and teeth [6].

Metal ion binding proteins, i.e., metalloproteins, play critical roles in a biological and chemical process in cellular reactions [7]. Inside the cell, the dynamic homeostasis of the metal ions is strongly connected and delicately tuned [8]. Reinhard et al. claimed that K^+^ and Na^+^ are involved in processing cell signaling, intercellular communication, and maintaining tissue electrolyte balance [9]. A small change in the concentration of metal ions may shift the effects of metal ions from natural beneficial to harmful [10]. A recent study pointed out that metalloproteins are associated with degenerative diseases, including Parkinson's disease and Alzheimer's disease [11]. For instance, *α*-synuclein (Cu^2+^-protein complex) constitutes the main component in Lewy bodies in Parkinson's disease [11]. Mn^2+^ and Fe^3+^ are responsible for inducing tangle pathology in Alzheimer's disease [8]. The aging of the brain or the development of diseases is associated with the deregulation of the management of metal ions [10]. Particularly, recent evidence indicates that if different types of metalloproteins interact in a certain salt solution, the potential galvanic erosion may dissolute the compound surface and result in inducing tumor formation [12, 13].

Elucidated protein-metal ion interactions rely in part on the advancement of various accurate characterizations and predictions of metalloproteins at the residue level. The traditional methods that are used to identify metal-binding conformation or binding residues include biophysics- or biochemistry-related wet-lab experiments, such as mass spectrometry [14], X-ray crystallography [15], and surface Plasmon resonance [16]. Since these technologies need expensive instruments, complex procedures, and elaborate labors, they shall benefit from the recent development of computation-aided methods.

We found 12 reviews that focused on the topic of exploring metal-binding residues or proteins in the past decade [7, 10, 11, 17–24]. Mallick et al. shed light on in silico methods including nine predictive tools and discussed the intrinsic mechanisms of metal-protein binding [24]. Thirumoorthy et al. investigated metallothionein isoforms and their role in pathophysiology [17]. They also provided the analysis of how metallothionein impact complex disease scenarios. In [18], the authors focused on structural variability and corresponding mechanisms of polymorphic amyloid oligomers complexed with metal ions. Bal et al. discussed ability constants, dissociation rates, and coordination chemistry of metal-binding residues in albumin [19]. Roohani et al. reviewed the literature related to zinc biochemical and physiological functions, metabolism, and zinc bioavailability in the human body [20]. The authors in [21] summarized the web tools that were proposed to identify metal-binding residues. Liu et al. systematically analyzed the structural features of Zn^2+^-binding sites and proposed an online predictor [22]. Akcapinar and Sezerman collected and surveyed computational toolboxes designed for the recognition of metal-binding sites or metalloproteins [7]. Quintanar and Kim summarized the research in degenerative diseases related in metal ions [11]. Witkowska and Rowińska-Żyrek overviewed the analytical and biophysical methods utilized for studies on metal-protein interactions [23]. Krzywoszyńska detailed the involvement of metal ions in signaling processes within the cell and its influence in health and disease [10]. Rauer et al. scrutinized computational approaches that are associated with the prediction of protein functional sites and also discussed metal-binding related works [25].

Broadly speaking, these reviews discuss some aspects of the predictive methods. Some of them provide sufficient coverage of databases and predictive models and discuss the challenges and limitations of considered approaches. These reviews bring informative clues for the following researchers in this field. From the pertinence of the research, the prediction of metal-binding can be divided into general and specific approaches. The former recognizes metal-binding residues without considering their types, while, the latter is aimed at identifying one or several specific metal-protein interactions. According to the basic design and scheme, we classify these methods into four categories, namely, learning-, docking-, template-, and meta-based methods.

This review covers a comprehensive set of 44 computation-based methods, and 25 of them were published in the past three years. Specifically, we survey 32 learning-based, 4 docking-based, 6 template-based, and 2 meta-based methods. Depending on whether the structure of a target protein is known or available, we further divide learn-binding methods into the structure- and sequence-based ones. We discuss their benchmark datasets, features, algorithms, and measurements, respectively. We also detail the docking-, template-, and meta-based methods and point out their advantages and limitations.

## 2. Public Databases for Metal Binding

**Figure 1:**
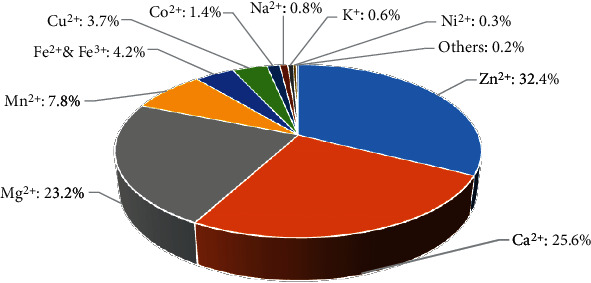
Fraction of top 10 metal-binding interactions that stored in PDB (date: December 20, 2021).

The development in biochemistry and biophysics leads to a fast increasing number of protein-metal ion binding complexes.  Figure 1 draws the top 10 metal-binding annotations in PDB. Our survey reveals that Zn^2+^, Ca^2+^, and Mg^2+^ occupy the top three prevalent metal ions. The Zn^2+^ is currently the best-explored and described metal ion [26]. Zn^2+^ participates in many biological processes, such as metabolism, immune system, neurotransmission, hormone secretion, and signaling [27]. According to a rough statistic, approximately 10% of eukaryotic proteins bind Zn^2+^ [28]. Ca^2+^ is mainly aggregated in bones and teeth vertebrates [29]. It helps form solid support structures through biomineralization [6]. Mg^2+^ is usually associated with solvent water molecules, which endow it with a good capability of binding affinity with proteins and movement. The solvation state of Mg^2+^ usually serves as the enzyme in which Mg^2+^ acts as a coenzyme [6].

Besides RCSB PDB (https://www.rcsb.org/) [30], recent years have witnessed several specific databases that collect, categorize, and store these metal-protein interactions.  Table 1 summarizes the publication year, considered metal ions, size of the database, web link, citations, and availability for the recently released database. We use citations as a one direct and good way to quantify the impact of these resources within the community [31]. The citation counts were collected from Google Scholar (https://scholar.google.com/) on December 20, 2021.

Specifically, InterMetalDB collects and presents metal ion binding proteins from RCSB PDB. It uses MMseq2 [32] to cluster the structure chains with the 50% sequence identity. Then, it groups similar binding sites and selects the best-resolution structure as a representative. MeLAD is a metalloenzyme-ligand association database, which contains structural data, metal-binding pharmacophores, and ligand chemical similarity of metalloenzyme-ligand interactions [33]. MetalPDB details the local environment, three-dimensional (3D) structure, secondary structure, and solvent accessibility of the metal ion binding sites [34, 35]. BioLiP is a semimanually curated database, which includes protein-peptide, protein-nucleic acid, and protein-ligand annotations [36]. BioLiP stores and periodically updates all types of metal ion binding information from PDB. ZiFDB is a database that collects information about individual zinc fingers, engineered zinc-finger arrays, and related target sequences [37, 38]. BioMe provides a web interface for biologists to capture coordination numbers, distances, geometry, and percentage of monodentate and bidentate bound aspartic acid and glutamic acid carboxyl groups [39]. MetLigDB is specially designed to select chelating groups or chemical moieties that might be presented in the inhibitor of a metalloprotein [40]. MIPS stores the geometric information, macromolecular function, different chemical behavior of metals, and metalloproteins [41]. MEDB presents quantitative information on metal-binding sites in protein structures and can be used for the identification of trends or patterns in the metal-binding sites [42]. MetalMine automatically collects and categorizes different types of metal-binding sites that derived from the structures of protein-metal-ion complexes [43]. Metal-MACiE gathers all available metalloenzymes and includes structural and functional information of metal ions in the context of the catalytic mechanisms of these metalloenzymes [44]. ZifBASE deposits engineered and natural zinc finger proteins and provides sequences and structural features and associated potential target sites of these proteins [45]. MESPEUS [46] focuses on the geometry of metal sites in proteins at resolution ≤ 2.5 Å. It provides an open web interface for further identifying and displaying the metal sites. MSDsite deposits computation-based metal-binding geometries and residues [47]. MDB offers quantitative information about metalloproteins [48]. It provides functions to analyze the binding attributes such as metal-ligand bond distances and side-chain torsion angles in metal sites.

We show that twelve source databases are designed for all metal ion binding data. Two databases, namely, MetLigDB and MESPEUS, consider several types of metal ions. There are four specific zinc-binding-related databases. Our survey also reveals that BioLiP is the most favored database, given the fact that its citations are average about 56 (446/8≈56) per year. Moreover, we notice that only half of the databases are available. Thus, we recommend that future databases shall be chronically maintained, periodically updated, and easy expanded.

## 3. Method Development of Metal-Binding Prediction

**Figure 2:**
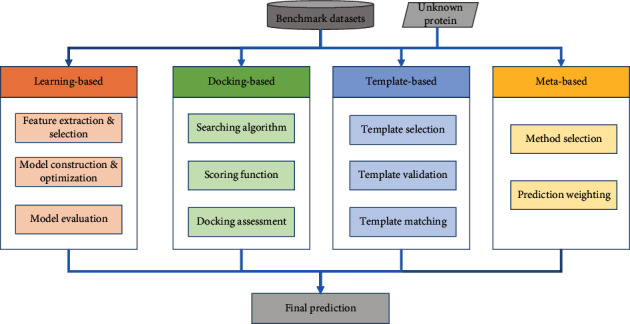
The flowchart of computation-based methods for prediction of metal-binding residues.

Figure 2 illustrates the flowchart of computation-based methods for the prediction of metal-binding residues. Generally, based on the basic design and scheme, these methods can be categorized into four groups. The learning-based methods regard the identification of metal-binding residues as a typical classification problem and attempt to use machine learning or deep learning algorithms to construct prediction models. The docking-based approaches are aimed at finding proper binding conformation as well as the appropriate target binding residues by scanning protein surface. The scoring functions are introduced to assess the selected pockets and quantify the strength of binding affinity. The template-based methods are designed to select the optimal template structures for a given unknown protein. Then, they map and transfer the binding annotations from similar spatial conformation to the target protein. By contrast, the meta-based methods focus on combing the predictions from other methods in order to build more accurate predictors.

### 3.1. Benchmark Datasets

The sequences and structures of protein-metal ion complexes are available in public databases for the end-users to customize the benchmark datasets. As shown in Table 2, the considered methods use various numbers of sequences/chains, ranging from several dozens to thousands. Besides that, protein complexes with high resolution indicate relatively more comprehensive and accurate annotations of protein-metal ion interactions. According to our survey, 12 out of 23 sequence-based and 6 out of 9 structure-based methods filter the candidate complexes using high resolution with ≤3 Å. Some methods [50–57] remove the sequences/chains whose lengths are less than 50 residues (or 45 residues [58]) since they might be potential segments or peptides. To build an unbiased dataset, it is necessary to remove homologous or redundant proteins. The cutoff threshold which researchers choose varies from minimal 25% to maximal 90%. Generally, a higher identity means a higher chance in local alignments [59]. The literatures in [60, 61] point out that if a pair of proteins have a sequence identity lower than 30%, they have little chance to share the same biological processes. Three tools, namely, BLASTclust [62], PISCES [63], and CD-HIT [64], are mainly used to cluster homologous proteins.

### 3.2. The Validation and Evaluation Metrics

#### 3.2.1. Cross-Validation and Independent Test

To construct a predictor with high accuracy and decent generalization ability, it is necessary to avoid potential overfitting. In practice, cross-validation and independent test are two popular ways (Table 2) to evaluate the proposed models [31]. Specifically, *k*-fold cross-validation is usually adopted on the training dataset when building the prediction model and optimizing the related parameters [61]. First, the training dataset is equally divided into *k* parts. The division can be done at residue level or protein level. Next, *k*-1 subsets are used to train the model, and the last one subset is used for testing. The procedure repeats *k* times until every subset is been predicted. The performance of the model is usually evaluated by averaging the results of the *k* repeats.

#### 3.2.2. Performance Measures

According to Table 2, the measures that used to evaluate the performance of the predictors can be divided into binary value-based and propensity score-based ones. The former needs preset thresholds to compute the number of putative binding residues and nonbinding residues. These measures include sensitivity (SN)/recall/true positive rate (TPR), specificity (SP), false positive rate (FPR, FPR = 1-SP) precision (PRE), accuracy (ACC), F1-score (F1), and Matthew's correlation coefficient (MCC). They are defined as follows:

(1) $$\begin{array}{c} \text{SN}=\text{TPR}=\frac{\text{TP}}{\text{TP}+\text{FN}},\\ \text{SP}=1-\text{FPR}=\frac{\text{TN}}{\text{TN}+\text{FP}},\\ \text{PRE}=\frac{\text{TP}}{\text{TP}+\text{FP}},\\ \text{F}1=\frac{2\text{TP}}{2\text{TP}+\text{FP}+\text{FN}},\\ \text{ACC}=\frac{\text{TP}+\text{TN}}{\text{TP}+\text{FN}+\text{TN}+\text{FP}},\\ \text{MCC}=\frac{\text{TP}\times \text{TN}-\text{FN}\times \text{FP}}{\sqrt{\left(\text{TP}+\text{FN}\right)\times \left(\text{TP}+\text{FP}\right)\times \left(\text{TN}+\text{FP}\right)\times \left(\text{TN}+\text{FN}\right)}}, \end{array}$$

where TP (true positive) indicates the number of correctly recognized metal-binding residues, FP (false positive) means the number of non-metal-binding residues that are incorrectly predicted as metal-binding residues, TN (true negative) stands for the number of correctly predicted non-metal-binding residues, and FN (false negative) is the number of metal-binding residues that are incorrectly predicted as non-metal-binding residues.

The prediction of metal-binding residues is a typical imbalanced classification problem. That is, the number of metal-binding residues is much less than that of the non-metal-binding ones. Therefore, F1-score and MCC are regarded as key criteria since they are featured by assessing the prediction performance for both metal-binding and non-metal ion binding residues.

The propensity score-based measures include receiver operating characteristic curve (ROC curve) and precision-recall curve (PR curve). The ROC curve draws the TPR (true positive rate) against the FPR (false positive rate) at various thresholds. The AUC computes the area under the ROC curve and can be used to quantify the ROC curve. The PR curve plots PRE values on the *y*-axis and recalls values on the *x*-axis, and the AUPRC estimates the area under the PR curve.

### 3.3. Learning-Based Methods

Learning-based methods treat the recognition of metal-binding residues as a typical pattern recognition problem. Specifically, the metal-binding residues and nonbinding ones are encoded by using mathematical descriptors, i.e. features. According to the information that used to compute the features, the learning-based methods can be further categorized into sequence-based and structure-based methods. The former only needs simple protein sequences to extract features when encoding the binding residues. These features include sequence directly derived, evolutionary profile-based, and putative structure-based features, while the latter uses both sequence and native structure data to mathematically describe a binding residue. We make a comprehensive literature search and collect 23 sequence- and 9 structure-based methods that were published after the year 2010.

#### 3.3.1. Feature Construction

**Figure 3:**
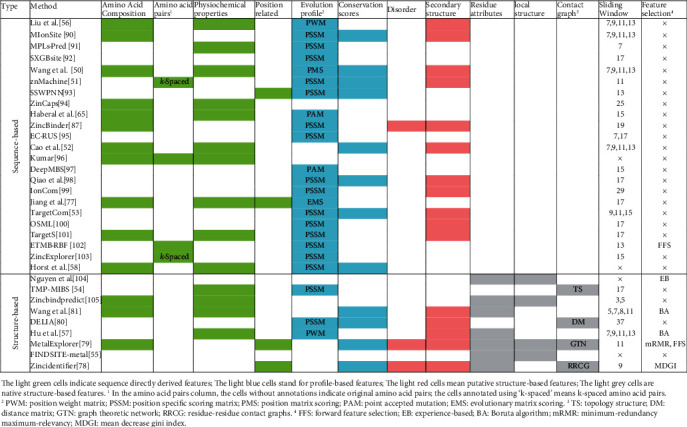
Summary of the feature construction and selection for learning-based methods. The light green cells indicate sequence directly derived features. The light blue cells stand for profile-based features. The light red cells mean putative structure-based features. The light grey cells are native structure-based features. ^1^In the amino acid pair column, the cells without annotations indicate original amino acid pairs; the cells annotated using “*k*-spaced” means *k*-spaced amino acid pairs. ^2^PWM: position weight matrix; PSSM: position specific scoring matrix; PMS: position matrix scoring; PAM: point accepted mutation; EMS: evolutionary matrix scoring. ^3^TS: topology structure; DM: distance matrix; GTN: graph theoretic network; RRCG: residue-residue contact graphs. ^4^FFS: forward feature selection; EB: experience-based; BA: Boruta algorithm; mRMR: minimum-redundancy maximum-relevancy; MDGI: mean decrease Gini index [50–51, 53–58, 65, 77–81, 87, 90–105].

*(1) Sequence Directly Derived Features*. We define sequence directly derived features as the ones that are computed from protein primary sequences without using any other information. In Figure 3, 14 out of 32 considered methods consider amino acid composition [50], which quantifies the relative difference in abundance of a given amino acid type [65, 66]. Amino acid pairs, or dipeptides, are based on the observation that amino acid pairs show different propensities in protein structure and function. For instance, pairs of lysine are found present in close spatial vicinity [67]. Moreover, the concept of *k*-spaced amino acid pairs is introduced in [68]. It calculates the amino acid pairs with *k* spaces between two residues. Our survey also shows that the majority of studies use physicochemical properties to describe the local environment of the metal-binding residues. The basic physicochemical environment of a metal-protein binding interface is reflected by the specific roles the metal plays in biostructural chemistry and protein function. These properties are crucial since they underpin many of the functional roles of metal ions. These properties include aliphatic [69], sulphur [70], aromatic [71], hydrophobic [72], charge [73], polar [74], positive [73], acidic [75], and hydroxylic [76]. The position-related features mainly consider the influence of the specifically located residues, such as autocross covariance [77] and sequence length [78, 79].

*(2) Evolutionary Profile-Based Features*. Recent studies [54, 56, 57, 80, 81] pointed out that functional or structural important residues tend to show higher evolutionary conservation. The conserved residues are usually involved in enzyme activity, ligand binding, or protein structural stability [82]. The conserved residues and regions can be identified by multiple sequence alignment [83]. These multiple sequence alignments, also named conservation profiles, include aligning families of homologous sequences and having knowledge of their evolutionary relationships [84]. For an unknown protein, although its accurate function is not available, it is expected that we can use its homologous proteins to speculate the function since they share the similar evolutionary profile [85]. Many studies use position-specific scoring matrix (PSSM), which is computed from PSI-BLAST [62], to quantify the evolutionary conservation. PSSM scores the substitution probability of each residue in the protein being substituted by other types of amino acids. Liu et al. [56] and Hu et al. [57] set different weights according to the positions of considered residues within the window and construct position weight matrix (PWM). Wang et al. proposed a customized position matrix scoring (PMS) algorithm, which uses known sequence patterns to describe the composition of amino acids at different positions [50]. Haberal and Oğul introduced a point accepted mutation (PAM) scoring matrix, which measures the rate at which point mutations that substitute one residue for another during evolution [65]. Jiang et al. adopted evolutionary matrix scoring (EMS) algorithm to extract the position conservation of amino acid residues from segments with low dimension feature parameters [77].

*(3) Putative Structure-Based Features*. For an unknown protein, although the accurate function is not available, it is expected that we can use its homologous proteins or template structures to speculate the structure. The secondary structure mainly involves *α*-helix, *β*-sheet, and coil, which are fundamental elements of protein tertiary structure [86]. Natively disordered or unstructured regions are proved to be associated with molecular assembly, protein translation, modification, and molecular recognition [78, 79, 87]. Previous studies [79, 87] indicate that disordered regions are strongly correlated with local solvent accessibility areas.  Figure 3 reveals that 16 methods introduce secondary structure features and 3 approaches use disorder features, respectively. The secondary structure can be obtained from the primary sequence by using PSIPRED [88]. Putative intrinsic disorder data can be computed by using DISOPRED [89].

*(4) Structure-Based Features*. The structure-based features include descriptors that are computed from protein 3D structure. These features include solvent exposure, B-factor, spatial cluster properties, and native secondary structure. Compared with the abovementioned putative structure-based features, the native structure-based features are more accurate since they are directly computed by using residue coordinate data. Besides that, a residue contact network is also considered by some literature. In [79], two residues are defined as being in contact if the distance of their C*α* atoms is less than a predefined cutoff distance of 6.5 Å. These features include clustering coefficient, degree, density, distance, topology structure, and graph theoretic network [55, 79, 80].

#### 3.3.2. Sliding Window Optimization and Feature Selection

As shown in Figure 3, many methods adopt a sliding window scheme when they construct different types of features. It is because residues in proteins are influenced by adjacent residues. Besides that, binding residues tend to cluster together. If a central residue is a native-binding residue, its adjacent residues usually have a relatively higher chance to bind the same ligands. Usually, the residues with a long distance away have a lower impact on the central residues when compared with the residues with short distance.  Figure 3 summarizes that 19 out of 23 methods use the sliding window scheme. The size of the shortest window is 3 [105], while the size for the longest one is 25 [94]. Some studies [50, 52, 53, 56, 57, 81, 90, 105] use more than one type of window because they consider different types of metal-binding residues. A long window means the introduction of more features.

However, a bigger number of features do not absolutely mean a better prediction performance [106, 107]. The existence of potential “bad” features may interfere with the classifiers and cause unpredictable consequences [108]. The so-called “bad” features include irrelevant and redundant ones. To avoid their terrible influences, it is necessary to perform feature selection before training the model [109].  Figure 3 reveals that 6 out of 32 methods adopt feature selection before training the model. These feature selection approaches include forward feature selection [79, 102], experience-based [104], Boruta algorithm [57, 81], minimum-redundancy maximum-relevancy [79], and mean decrease Gini index [78].

#### 3.3.3. Prediction Algorithms

Learning-based methods use machine-learning or deep-learning-based algorithms to train the model and perform predictions [110]. As shown in Table 2, a variety of algorithms are introduced for solving the problem of correctly recognizing metal-binding residues. Support vector machine (SVM) is a popular machine learning algorithm in bioinformatical research. It is aimed at finding a hyperplane or decision boundary that can segregate a high-dimensional space [111]. Particularly, it uses kernel functions to reduce computation time to avoid strapping into dimension disaster [112]. Sequential minimal optimization (SMO) is an algorithm that is specially used for training support vector machines [113]. The procedure of training large data by SVM usually leads to a complex quadratic programming optimization problem [114]. SMO breaks large programming optimization problems into small ones, which endows SVM a good generalization on large data [113]. The idea of a neural network (NN) comes from the work system of neurons in the biological brain [115]. It learns the correlations between inputs and outputs, making generalizations and build models [116]. The NN algorithm assigns and adjusts different weights for neurons and edges as learning proceeds. The radial basis function network (RBFN) is a variant of the original NN [117]. It adopts radial basis functions as activation functions, which can be used for accelerating learning speed due to their universal approximation [118]. The multilayer perceptron (MLP) algorithm is an improved back propagation NN [119]. It mainly includes three procedures: forward propagation, error evaluation, and error backpropagation [120]. The MLP is featured by its strong generalization and fault tolerance [121]. Therefore, it is proved to be an efficient classification algorithm. The logistic regression (LR) adopts a logistic function to model the probability of an unknown sample being a certain class [122].

Our survey also reveals that the ensemble algorithms are favored by eight studies. The random forest (RF) aggregates the predictions of all the decision trees and performs decisions by most trees [123]. RF can be used for classification, regression, and optimization problems [124]. Adaptive boosting (AdaBoost) is aimed at combining weak learners with strong ones [125, 126]. The key point of AdaBoost is to ensure the diversities of individual learners, which makes it a good generalization ability [90, 127]. The gradient boosting machine (GBM) is another popular ensemble algorithm. During the iterative process, GBM dynamically increases the weight of wrong recognitions and reduces that of the correct ones [128–131]. It should be noted that GBM focuses on the sample residual of the previous iteration instead of the sample itself [132].

Besides machine-learning algorithms, recent studies also use deep-learning methods in this research field. The convolutional neural network (CNN) is one of the most prevalent algorithms that is widely used in bioinformatics [133]. The CNN consists of three main layers, which are the convolutional layer, pooling layer, and fully connected layer [134–136]. Although the CNN is proved to be powerful in dealing with a variety of problems, it performs badly when facing samples with different sizes and orientations [137, 138]. To overcome this shortcoming, the capsule network (CN) is proposed to estimate features of objects by incorporating dynamic routing algorithms [139, 140]. Our review finds two studies use CNN [65, 97] and one uses CN [94].

### 3.4. Docking-Based Methods

The investigation on the protein-metal complex helps biologists to understand the mechanism of protein-metal interactions. Protein-ligand docking approaches are always based on molecular structure and are used to explore biomolecular interactions and mechanisms [141]. It can be adopted to predict binding conformation as well as the appropriate target binding residues [142, 143].

**Figure 4:**
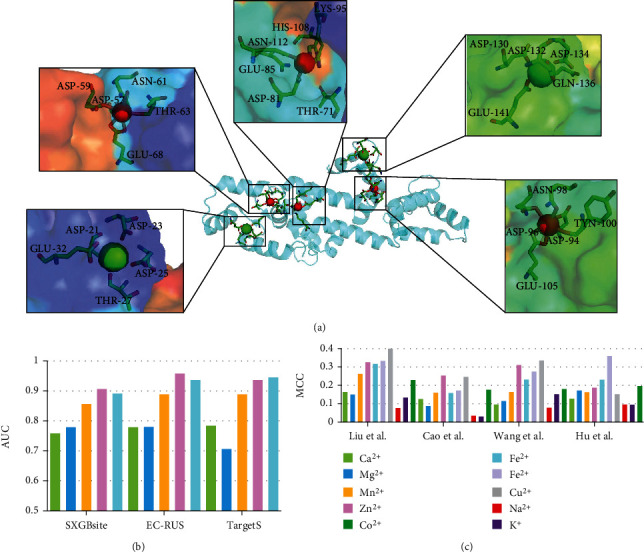
Ribbon and surface model of X-ray structure of Ca^2+^- and Zn^2+^-bound calmodulin (PDB: 4HEX) in *Mus musculus*. Red sphere represents bound zinc ion; green one indicates calcium ion; the spatial adjacent residues participating its coordination are shown by the stick model.

As shown in Figure 2, the docking-based methods mainly include three steps: searching algorithm, scoring function, and docking assessment [141]. The searching algorithm focuses on creating an optimum number of configurations that properly include the determined binding modes [144]. To reduce computation time, it is necessary to make a balance between the computational expense and the searching space. The scoring function includes a series of mathematical functions that quantify the strength of binding affinity [145]. The energy-based scoring functions are always introduced to score the potential interactions between the protein and the corresponding ligands [141]. The frequently used functions include empirical-based, knowledge-based, and consensus-based ones. Finally, the putative docking can be evaluated by using docking accuracy and the correlation between putative and native docking scores [145].  Figure 4 illustrates the structure of a calmodulin (PDB: 4HEX) that is secreted by *Escherichia coli* in *Mus musculus* [146]. Calmodulin is one of the most prevalent EF-hand calcium sensor proteins in eukaryotic cells [147]. It is a highly conserved and soluble protein, which activates enzymes and regulates many cellular functions. 4HEX has three Ca^2+^-binding and two Zn^2+^-binding sites. Ca^2+^-binding causes a change in calmodulin conformation opening both globular domains and exposing hydrophobic surfaces that form binding sites for the target enzymes.  Figure 4 shows that these three Ca^2+^ are in the pockets. The binding pockets are half-closed and buried, which substantially limits the capability of Ca^2+^ to escape. Two Zn^2+^-binding sites are surrounded by a shell of hydrophilic groups that are embedded into a larger shell of hydrophobic groups. The amino acid side chains providing ligands to Zn^2+^ in these structures often form hydrogen bonds with other residues [147].

In [148], He et al. proposed a docking-based predictor named mFASD. It first explored the local biochemical environment of potential functional atoms and then measured the distances between the atoms and bound metal. mFASD also claimed that it can differentiate different types of metal-binding sites. Zhou et al. improved the FEATURE-based calcium model and used the grid scan algorithm to recognize binding sites [149]. GaudiMM [150] adopted a multiobjective genetic algorithm to search metal-binding sites in biological scaffolds. BioMetAll focused on the conformation of the potential metal-binding site, associated with the geometric organization of the protein backbone [151]. It was also proved to have good performance on the applications including the modulation and mutation of the metal-binding residues.  Table 3 summarizes the key notes of the abovementioned 4 docking-based methods.

### 3.5. Template-Based Methods

It is well known that protein structure determines function, and similar interface conformation indicates similar bound regions [162]. The template-based methods are based on the abovementioned hypothesis. Therefore, the most important thing for template-based methods is to find and validate proper structural templates. The fold recognition algorithms, which quantify the best matches from candidate templates, are commonly used to select the optimal template structures [163]. Next, the selected templates are used to map onto the target protein given the alignments with the template structures [164].

As shown in Table 3, Goyal and Mande analyzed the metal-binding sites by using structure templates and designing 3D motifs for several types of metal-binding interactions [153]. In [154], the authors analyzed whether structural models based on remote homology are effective in recognizing structural metal-binding residues based on simple protein primary sequences. Deng et al. applied a graph theory algorithm to identify, predict, and analyze calcium-binding residues [152]. However, it should be noted that this strategy produces good prediction performance when a decent complex is available as a template. If the template structure information is not available, this strategy might have poor predictions [164]. The FunFOLD was an automatic method that uses protein structure superposition of distantly related templates to a modelled protein for the clustering of ligands and prediction of metal binding residues [155]. The FunFOLDQA [156] approach determined the reliability of our FunFOLD [155] by assigning the quality assessment scores. FunFOLD2 was a web server that integrated cutting edge function and putative 3D structures to identify metal-binding residues [157].

### 3.6. Meta-Based Methods

The meta-based methods use a meta-learning strategy from fewer samples than traditional machine learning models. Since meta-based methods can only use limited data, they must ensure that the data is featured with high accuracy. As a result, a meta-based approach always directly combines the predictions of other methods. It uses weights or voting strategy on the available propensity scores or binary values. Thus, the meta-based method promises a robust accurate prediction on the metal-binding residues. In [158], Li et al. collected the predictions from ZincExplorer [103], ZincFinder [159], and ZincPred [160] (Table 3). Then, they built a linear regression model and optimized corresponding parameters on the training dataset. They claimed that the meta-model, which was named meta-zincPrediction, improves the AUPRC by about 2%~8%. IBayes_Zinc [161] was another meta-based predictor for the identification of zinc-binding residues (Table 3). It firstly computed the predictions of zinc-binding probabilities from ZincExplorer [103], ZincFinder [159], and ZincPred [160]. Next, IBayes_Zinc processed the missing attribute values and adopts Bayesian theory [165] to construct a meta-based model. The performance on the independent dataset proved that the MCC value of IBayes_Zinc was about 5~13% higher than the considered three predictors.

### 3.7. Prediction Results

This review surveys 44 computation-based methods. It is necessary to make a consensus comparison for these methods. However, since there is no standard benchmark dataset and some methods are not currently available, we use two datasets that are used by some methods to perform the evaluations. The first dataset is compiled by Yu et al. in [101], which includes five types of metal ions binding annotations. The second dataset is obtained from [52], consisting of ten types of metal ions binding annotations.

**Figure 5:**
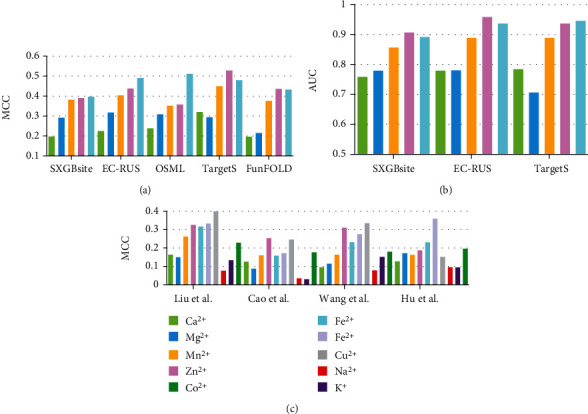
Comparative assessment of several predictors on two benchmark dataset. (a) and (c) indicate the MCC bar charts for considered methods on different metal ion binding residues on Yu et al.'s and Cao et al.'s testing datasets, respectively. (b) draws the AUC values of three predictors on corresponding metal ion binding residues.

Figure 5 illustrates the predictive performance on two benchmark test datasets. Details are provided in Table S1 and Table S2 in Supplementary Materials, respectively; the corresponding results are sourced from [56, 57, 81, 92]. We notice that the predictors show relatively big differences in recognizing various types of metal-binding residues. On Yu et al.'s dataset [101], TargetS shows the best results in predicting Ca^2+^-, Zn^2+^-, and Mn^2+^-binding residues; EC-RUS [95] performs best in recognizing Mg^2+^-binding residues; OSML [100] achieves the highest MCC on Fe^3+^-binding predictions. Besides that, Figure 5(a) indicates that all five methods show a decent performance on recognizing Fe^3+^-binding residues (MCC values close or higher than 0.4), compared with MCC close or less than 0.2 on Ca^2+^ binding residues.  Figure 5(b) draws the bars of AUC values for SXGBsite [92], EC-RUS [95], and TargetS [101], respectively. These three predictors all achieve high AUC scores (close or higher than 0.9) on Zn^2+^- and Fe^3+^-binding residues.  Figure 5(c) summarizes the results of ten metal ions binding residues on Cao et al.'s dataset [52]. Among these predictors, Liu et al. [56] performs the best on Zn^2+^, Fe^3+^, and Cu^2+^, compared to [81], Wang et al. shows best on Zn^2+^ and Cu^2+^, and Hu et al. [57] achieves the highest on Fe^2+^. Interestingly, the binding residues associated with relatively inactive metal (Zn^2+^, Fe^3+^, and Cu^2+^) ions show relatively better results compared to that of the active metal ions (Na^+^ and K^+^). Particularly, four methods all give better results on Fe^3+^-binding residues than that on Fe^2+^-binding residues, which keep consistent with our observations as mentioned above.

### 3.8. Publicly Available Tools

The publicly available standalone software or web server that implements the proposed approach provides convenience for biologists and researchers [79, 105, 122]. These tools help the community to repeat the results and build a platform for easy understanding and improvement.  Table 4 summarizes the public availability of implementations for the considered methods. These 28 predictors are implemented as standalone software or web servers. Among these predictive tools, 16 (or 57%) of them are currently publicly available. Standalone software requires the biologists to build the same running environment. By contrast, the web server provides the most convenient since the users only need to submit their queries via the browser, and the server helps to do the computations. Three methods, namely, znMachine [51], ZincBinder [103], and FunFOLD [155], provide both web server and standalone software. TMP_MIBS [54] is designed to predict general metal-binding residues and deployed using the Python language. DELIA [80] requires PDB-formatted 3D coordinates input and produces both binary prediction and putative probability of a residue being potential specific metal-binding. BioMetAll uses a docking-based strategy to scan specific motifs, putative mutations, and binding residues. Another available docking-based method is mFASD, which distinguishes different types of metal-binding sites according to the interaction distances. MPLs-Pred [91], SXGBsite [92], MIonSite [90], OSML [100], EC-RUS [95], and TargetS [101] are all sequence-based predictive tools, which accepts FASTA-formatted input and produced the results of putative metal-binding residues. ZinCaps [94], SSWPNN [93], and ZincBinder [103] are specially designed for the identification of zinc-binding residues. FunFOLD [155], FunFOLDQA [156], and FunFOLD2 [157] are a series of template-based methods.

## 4. Conclusions and Future Perspectives

This review summarizes the public database of metal ions binding interactions, discusses the architectures of computation-based methods for identifying binding residues, and comparatively evaluates four types of methods. Based on the observations made in this work, we propose a few recommendations for future research in this field:

*First*, the researchers should maintain and update the database regularly. This will significantly improve effectiveness and completeness for these databases and provide convenience for the computation-based methods, which depend on the accurate internal database. We expect a high-quality metal ion binding-related database with an advanced searching engine, high-speed download service, complete annotation information, etc. Particularly, a decent database should be designed to open for easy expanding and improvement. *Second*, standard benchmark datasets that related to general or ligand-specific metal-binding residues should be periodically compiled and made available. This will ensure consistent evaluation and comparative analysis of the performance of the existing and novel methods. *Third*, these predictors are expected to use delicate architectures and powerful algorithms. Since the differences between different types are quite small, the novel predictors shall not only correctly identify metal-binding residues but also distinguish different types of metal ions. *Fourth*, the authors of the metal-binding predictors are suggested to make their approaches publicly available, preferably as both webservers and standalone software. Particularly, high-throughput predictors promise a wide application among the research community since they can be used to perform large-scale computations, such as proteome-level predictions.

## Figures and Tables

**Table 1:** Summary of recently released database of metal ion binding interactions.

Name	Year	Considered metal ions	Number of sites	Web link	Ref.	Citation	Availability
InterMetalDB	2021	All metal ion binding	6,423	https://intermetaldb.biotech.uni.wroc.pl/	[26]	N/A	Yes
MeLAD	2020	All metal ion binding	N/A	https://melad.ddtmlab.org/	[33]	9	Yes
ZincBindDB	2019	Zn	24,992	https://github.com/samirelanduk/ZincBindDB	[49]	23	Yes
MetalPDB (v2)	2018	All metal ion binding	N/A	http://metalweb.cerm.unifi.it	[34]	90	No
BioLiP	2013	All metal ion binding	146,969	https://zhanggroup.org/BioLiP/	[36]	446	Yes
ZiFDB (v2)	2013	Zn	N/A	http://bindr.gdcb.iastate.edu/ZiFDB	[37]	25	No
MetalPDB (v1)	2013	All metal ion binding	N/A	http://metalweb.cerm.unifi.it	[35]	108	No
BioMe	2012	All metal ion binding	20,307	http://metals.zesoi.fer.hr	[39]	30	No
MetLigDB	2011	Zn, Mn, Fe, Ni, mg, cu, co, Mo	732	http://silver.sejong.ac.kr/MetLigDB	[40]	13	Yes
MIPS	2010	All metal ion binding	N/A	http://dicsoft2.physics.iisc.ernet.in/mips/	[41]	28	Yes
MEDB	2010	All metal ion binding	N/A	http://www.uohyd.ernet.in/anambs/	[42]	14	No
ZiFDB (v1)	2009	Zn	N/A	http://bindr.gdcb.iastate.edu/ZiFDB	[38]	87	No
MetalMine	2009	All metal ion binding	412	http://metalmine.naist.jp	[43]	3	No
Metal-MACiE	2009	All metal ion binding	N/A	https://www.ebi.ac.uk/thornton-srv/databases/Metal_MACiE/home.html	[44]	60	Yes
ZifBASE	2009	Zn	N/A	https://web.iitd.ac.in/~sundar/zifbase/	[45]	35	Yes
MESPEUS	2008	Na, mg, K, ca, Mn, Fe, co, Ni, cu, Zn	34,896	http://eduliss.bch.ed.ac.uk/MESPEUS/	[46]	102	No
MSDsite	2005	All metal ion binding	N/A	http://www.ebi.ac.uk/msd-srv/msdsite	[47]	122	Yes
MDB	2002	All metal ion binding	N/A	http://metallo.scripps.edu/	[48]	276	No

^1^We estimate the availability on December 1st, 10th, and 20th of 2021, respectively.

**Table 2:** Summary of learning-based methods.

Type	Method^1^	Ref.	Year	Metal ion binding^2^	Dataset^3^	Resolution	Sequence similarity (tool)^4^	Prediction model^5^	Cross-validation	Independent test	Measurements^6^
Sequence-based	Liu et al.	[56]	2020	Zn, Cu, Fe, Co, Mn, Ca, Mg, Na, K	5,340	≤3 Å	30% (CD-HIT)	RF	5-fold	√	SN, SP, ACC, MCC
	MIonSite	[90]	2019	Zn, Ca, Mg, Mn, Fe, Cu, Fe, Co, Na, K, Cd, Ni	7,676	N/A	30% (CD-HIT)	SVM, AdaBoost	5-fold	√	SN, SP, ACC, MCC, AUC
	MPLs-Pred	[91]	2019	General metal ions	1,492	N/A	30% (CD-HIT)	RF	10-fold	√	SN, SP, ACC, MCC
	SXGBsite	[92]	2019	Ca, Zn, Mg, Mn, Fe	4,421	N/A	40% (PISCES)	GBM	5-fold	√	SN, SP, ACC, MCC, AUC
	Wang et al.	[50]	2019	Zn, Cu, Fe, Mn, Ca, Mg, Na, K	5,146	≤3 Å	30% (N/A)	SVM, SMO	5-fold	√	SN, SP, ACC, MCC
	znMachine	[51]	2019	Zn	2,043	≤3 Å	30% (BLASTclust)	SVM, NN	5-fold	√	SN, SP, ACC, MCC, PRE, AUC
	SSWPNN	[93]	2019	Zn	213	≤2.5 Å	70% (N/A)	SVM, NN	5-fold	√	SN, SP, PRE, F1, MCC, ACC
	ZinCaps	[94]	2019	Zn	738	≤3 Å	N/A (N/A)	CN	5-fold	√	SN, SP, ACC, MCC, AUC
	Haberal and Oğul	[65]	2018	General metal ions	2,727	N/A	N/A (N/A)	CNN	5-fold	√	SN, ACC, PRE, F1
	ZincBinder	[87]	2018	Zn	738	≤2.5 Å	30% (PISCES)	SVM	5-fold	√	SN, SP, ACC, MCC, AUC
	EC-RUS	[95]	2017	Ca, Mg, Mn, Fe, Zn	4,421	N/A	40% (PISCES)	WSRC	5-fold	√	SN, SP, ACC, MCC, AUC
	Cao et al.	[52]	2017	Zn, Cu, Fe, Co, Mn, Ca, Mg, K, Na	5,340	≤3 Å	30% (CD-HIT)	SVM	5-fold	√	SN, SP, ACC, MCC
	Kumar	[96]	2017	Cu, Ca, Co, Fe, Mg, Mn, Ni, Zn	3,922	N/A	50% (CD-HIT)	RF	10-fold	√	SN, SP, ACC, MCC
	DeepMBS	[97]	2017	General metal ions	2,727	≤3 Å	N/A (N/A)	CNN	5-fold	√	SN, PRE, F1
	Qiao et al.	[98]	2017	Ca	2,239	N/A	30% (CD-HIT)	SVM	5-fold	√	SN, ACC, PRE, MCC, AUC
	IonCom	[99]	2016	Zn, Cu, Fe, Ca, Mg, Mn, Na, K	1,374	N/A	30% (CD-HIT)	SVM, AdaBoost	5-fold	√	SN, SP, ACC, MCC
	Jiang et al.	[77]	2016	Ca	1,885	≤3 Å	25% (N/A)	SVM	5-fold	√	SN, SP, ACC, MCC
	TargetCom	[53]	2016	Cu, Fe, Zn	1,373	≤3 Å	40% (CD-HIT)	SVM, AdaBoost	5-fold	√	SN, SP, ACC, MCC
	OSML	[100]	2015	Ca, Zn, Mg, Mn, Fe	4,421	N/A	40% (PISCES)	SVM	5-fold	√	SN, SP, ACC, MCC
	TargetS	[101]	2013	Ca, Zn, Mg, Mn, Fe	4,421	N/A	40% (PISCES)	SVM, AdaBoost	5-fold	√	SN, SP, ACC, MCC, AUC
	ETMB-RBF	[102]	2013	General metal ions	55	N/A	20% (BLASTclust)	RBFN	10-fold	√	SN, SP, ACC, MCC
	ZincExplorer	[103]	2013	Zn	392	≤3 Å	N/A (N/A)	SVM	5-fold	√	SN, SP, PRE, MCC, AUPRC
	Horst et al.	[58]	2010	Ca	635	≤2.1 Å	35% (N/A)	LR	10-fold	√	MCC,AUC,AUPRC
Structure-based	Nguyen et al.	[104]	2021	Mn, Fe, Co, Ni, Cu, Zn	9,955	≤2.5 Å	90% (N/A)	RF	5-fold	×	ACC
	TMP-MIBS	[54]	2021	General metal ions	427	N/A	40% (CD-HIT)	RF	10-fold	√	SN, SP, ACC, MCC, AUC
	Zincbindpredict	[105]	2021	Zn	N/A	≤ 2 Å	40% (CD-HIT)	RF	5-fold	√	SN, PRE, F1, MCC
	Wang et al.	[81]	2021	Zn, Cu, Fe, Ca, Mg, Mn, Na, K, Co	5,340	≤3 Å	30% (N/A)	MLP,SVM	5-fold	√	SN, SP, ACC, MCC
	DELIA	[80]	2020	Ca, Mn, Mg	3,966	N/A	30% (CD-HIT)	CNN	5-fold	√	SN, PRE, MCC, AUC
	Hu et al.	[57]	2020	Zn, Cu, Fe, Co, Mn, Ca, Mg, Na, K	5,340	≤3 Å	30% (CD-HIT)	GBM	5-fold	√	SN, SP, FPR, ACC, MCC
	MetalExplorer	[79]	2017	Ca, Co, Cu, Fe, Ni, Mg, Mn, Zn	3,192	≤2.5 Å	30% (CD-HIT)	RF	5-fold	√	SN, FPR, PRE, AUC, AURPC
	FINDSITE-metal	[55]	2011	Ca, Co, Cu, Fe, Mg, Mn, Ni, Zn	860	N/A	35% (PISCES)	SVM	2-fold	√	ACC, SPC, PPV
	Zinc identifier	[78]	2011	Zn	1,103	≤2.5 Å	N/A (N/A)	RF	5-fold	√	SN, PRE, SP, FPR, AUC, AUPRC

^1^The name of each method is provided in either the publication or the last name of its first author. ^2^General metal ions mean that the related predictor does not differentiate the types of metal ion binding. Otherwise, we list the specific types of metal-binding in detail. ^3^The number represents the size of the benchmark dataset. ^4^The value reveals the protein similarity threshold in the benchmark dataset. The content in the blanket indicates the tool that is used for clustering proteins. ^5^SMO: sequential minimal optimization; SVM: support vector machine; WSRC: weighted sparse representation based classifier; NN: neural network; CN: capsule network; CNN: convolutional neural networks; RF: random forest; GBM: gradient boosting machine; RBFN: radial basis function networks; LR: logistic regression; MLP: multilayer perceptron. ^6^SN: sensitivity/recall; SP: specificity; ACC: accuracy; MCC: Matthew's correlation coefficient; PRE: precision; F1: F1-score; AUC: area under the ROC curve; AUPRC: area under the precision recall curve; FPR: false positive rate (FPR = 1-SP).

**Table 3:** Summary of docking-based, template-based, and meta-based methods.

Type	Method	Year	Notes
Docking-based	mFASD [148]	2015	Capture the characteristics of metal-binding sites and discriminate most types of these sites
	Zhou et al. [149]	2015	Use a FEATURE-based calcium model and convert high scoring regions into specific site predictions
	GaudiMM [150]	2019	Find poses that satisfy metal-derived geometrical rules and use post optimizations
	BioMetAll [151]	2020	Predict metal-binding sites with particular motifs, determine transient sites in structures, and predict potential mutations to generate convenient sites
Template-based	Deng et al. [152]	2006	Use a graph theory algorithm to find oxygen clusters of the protein (high potential for calcium binding)
	Goyal et al. [153]	2008	Describe generation of 3D-structural motifs for metal-binding sites from the known metalloproteins
	Levy et al. [154]	2009	Analyze whether structural models based on remote homology are effective in predicting 3D metal binding sites
	FunFOLD [155]	2011	Use an automated method for ligand clustering and identification of binding residues
	FunFOLDQA [156]	2012	Use a fully automated agglomerative clustering approach for both ligand identification and residue selection
	FunFOLD2 [157]	2013	Propose a method that include protein-ligand binding prediction and quality assessment protocol
Meta-based	Li et al. [158]	2017	Integrate the results of ZincExplorer [103], zincFinder [159], and zincPred [160]
	IBayes_Zinc [161]	2019	Adopt Bayesian method and combine the predictions from ZincExplorer [103], zincFinder [159], and zincPred [160]

**Table 4:** A breakdown of predictive tools of metal-binding residues.

Method	Year	Platform^1^	Web link	Availability^2^
TMP-MIBS [54]	2021	SS	https://github.com/QuJing785464/TMP_MIBS	Yes
Wang et al. [50]	2021	WS	http://39.104.77.103:8081/lsb/HomePage/HomePage.html	No
Zincbindpredict [105]	2021	WS	https://zincbind.bioinf.org.uk/predict/	No
DELIA [80]	2020	WS	http://www.csbio.sjtu.edu.cn/bioinf/delia/	Yes
BioMetAll [151]	2020	SS	https://github.com/insilichem/biometall	Yes
MPLs-Pred [91]	2019	WS	http://icdtools.nenu.edu.cn/	Yes
SXGBsite [92]	2019	SS	https://github.com/Lightness7/SXGBsite	Yes
MIonSite [90]	2019	SS	https://github.com/LiangQiaoGu/MIonSite.git	Yes
znMachine [51]	2019	WS&SS	http://bioinformatics.fzu.edu.cn/znMachine.html	No
ZinCaps [94]	2019	SS	https://github.com/clemEssien/ActiveSitePrediction	Yes
EC-RUS [95]	2017	SS	https://github.com/6gbluewind/protein_ligand_binding_site	Yes
MetalExplorer [79]	2017	WS	http://metalexplorer.erc.monash.edu.au/	No
Cao et al. [52]	2017	WS	http://60.31.198.140:8081/metal/HomePage/HomePage.html	No
ZincBinder [103]	2017	WS&SS	http://proteininformatics.org/mkumar/znbinder/	Yes
SSWPNN [93]	2017	SS	http://net.jitsec.cn:88/UploadedImages/SSWPNN.rar	Yes
Jiang et al. [77]	2016	WS	http://202.207.29.245/	No
TargetCom [53]	2016	SS	http://dase.ecnu.edu.cn/qwdong/TargetCom/TargetCom_standalone.tar.gz	No
OSML [100]	2015	WS	http://www.csbio.sjtu.edu.cn/OSML/	Yes
mFASD [148]	2015	SS	http://staff.ustc.edu.cn/liangzhi/mfasd/	Yes
FunFOLD2 [157]	2013	WS	http://www.reading.ac.uk/bioinf/FunFOLD/FunFOLD_form_2_0.html	Yes
ZincExplorer [103]	2013	WS	http://protein.cau.edu.cn/ZincExplorer	No
TargetS [101]	2013	WS	http://www.csbio.sjtu.edu.cn/TargetS/	Yes
FunFOLDQA [156]	2012	SS	http://www.reading.ac.uk/bioinf/downloads/	Yes
Zincidentifier [78]	2012	WS	http://protein.cau.edu.cn/zincidentifier/	No
FINDSITE-metal [55]	2011	WS	http://cssb.biology.gatech.edu/findsite-metal/	No
FunFOLD [155]	2011	WS&SS	http://www.reading.ac.uk/bioinf/FunFOLD/	Yes
Goyal et al. [153]	2008	WS	http://sunserver.cdfd.org.in:8080/protease/PAR_3D/index.html	No
Deng et al. [152]	2006	SS	http://chemistry.gsu.edu/faculty/Yang/GG.htm	No

^1^WS: web server; SS: standalone software. ^2^The availability was estimated on Dec 1st, 10th, and 20th of 2021, respectively.

## Supplementary Materials

Supplementary Materials Table S1: comparative assessment of considered methods on Yu et al.'s independent testing dataset. Table S2: comparative assessment of considered methods on Cao et al.'s independent testing dataset.
