# Supplementary material for: A Comprehensive Review of Computation-Based Metal-Binding Prediction Approaches at the Residue Level
Source: Biomed Res Int. 2022 Mar 31;2022:8965712. doi: 10.1155/2022/8965712 (PMC8989566; doi:10.1155/2022/8965712)
Supplement: Supplementary Materials — Table S1: comparative assessment of considered methods on Yu et al.'s independent testing dataset. Table S2: comparative assessment of considered methods on Cao et al.'s independent testing dataset. [file 8965712.f1.docx]

Supplement

Comprehensive review of computation-based metal-binding prediction approaches at the residue level

Nan Ye ^1,^ *, Feng Zhou ^2^, Xingchen Liang ^2^, Haiting Chai ^3^, Jianwei Fan ^2^, Bo Li ^4^, Jian Zhang ^2^

^1^ School of Finance and Economics, Xinyang Agriculture and Forestry University, Xinyang 464000, P.R. China;

^2^ School of Computer and Information Technology, Xinyang Normal University, Xinyang 464000, P.R. China;

^3^ College of Medical, Veterinary and Life Sciences, University of Glasgow, Glasgow, G12 8QQ, United Kingdom;

^4^ College of Electronic Science and Engineering, Jilin University, Changchun, 130012, China;

* Corresponding author: leavesyn@yeah.net

**Table S1**. Comparative assessment of considered methods on Yu et al.’s independent testing dataset.

| Method | Metal ion | SN | SP | ACC | MCC | AUC |
| --- | --- | --- | --- | --- | --- | --- |
| SXGBsite | Ca^2+^ | 0.133 | 0.997 | 0.987 | 0.197 | 0.758 |
|  | Mg^2+^ | 0.198 | 0.998 | 0.991 | 0.291 | 0.779 |
|  | Mn^2+^ | 0.342 | 0.995 | 0.988 | 0.382 | 0.856 |
|  | Fe^3+^ | 0.387 | 0.994 | 0.987 | 0.396 | 0.891 |
|  | Zn^2+^ | 0.410 | 0.992 | 0.986 | 0.390 | 0.906 |
| EC-RUS | Ca^2+^ | 0.173 | 0.996 | 0.987 | 0.225 | 0.779 |
|  | Mg^2+^ | 0.201 | 0.998 | 0.991 | 0.317 | 0.780 |
|  | Mn^2+^ | 0.358 | 0.996 | 0.989 | 0.403 | 0.888 |
|  | Fe^3+^ | 0.443 | 0.996 | 0.990 | 0.490 | 0.936 |
|  | Zn^2+^ | 0.489 | 0.992 | 0.986 | 0.437 | 0.958 |
| OSML | Ca^2+^ | 0.230 | 0.988 | 0.971 | 0.238 | N/A |
|  | Mg^2+^ | 0.338 | 0.987 | 0.976 | 0.308 | N/A |
|  | Mn^2+^ | 0.443 | 0.982 | 0.973 | 0.351 | N/A |
|  | Fe^3+^ | 0.529 | 0.990 | 0.982 | 0.511 | N/A |
|  | Zn^2+^ | 0.352 | 0.990 | 0.978 | 0.357 | N/A |
| TargetS | Ca^2+^ | 0.192 | 0.997 | 0.984 | 0.320 | 0.784 |
|  | Mg^2+^ | 0.183 | 0.998 | 0.988 | 0.294 | 0.706 |
|  | Mn^2+^ | 0.401 | 0.995 | 0.987 | 0.449 | 0.888 |
|  | Fe^3+^ | 0.483 | 0.993 | 0.987 | 0.479 | 0.945 |
|  | Zn^2+^ | 0.464 | 0.995 | 0.987 | 0.527 | 0.936 |
| FunFOLD | Ca^2+^ | 0.122 | 0.996 | 0.981 | 0.196 | N/A |
|  | Mg^2+^ | 0.220 | 0.991 | 0.983 | 0.215 | N/A |
|  | Mn^2+^ | 0.233 | 0.998 | 0.987 | 0.376 | N/A |
|  | Fe^3+^ | 0.472 | 0.991 | 0.984 | 0.432 | N/A |
|  | Zn^2+^ | 0.365 | 0.995 | 0.986 | 0.436 | N/A |

**Table S2**. Comparative assessment of considered methods on Cao et al.’s independent testing dataset.

| Method | Metal ion | SN | SP | ACC | MCC |
| --- | --- | --- | --- | --- | --- |
| Liu et al. | Ca^2+^ | 0.511 | 0.887 | 0.881 | 0.163 |
|  | Mg^2+^ | 0.746 | 0.818 | 0.817 | 0.150 |
|  | Mn^2+^ | 0.729 | 0.919 | 0.917 | 0.262 |
|  | Zn^2+^ | 0.922 | 0.907 | 0.907 | 0.326 |
|  | Fe^3+^ | 0.727 | 0.943 | 0.940 | 0.316 |
|  | Fe^2+^ | 0.790 | 0.937 | 0.935 | 0.333 |
|  | Cu^2+^ | 0.880 | 0.939 | 0.938 | 0.399 |
|  | Na^+^ | 0.543 | 0.728 | 0.725 | 0.076 |
|  | K^+^ | 0.872 | 0.512 | 0.523 | 0.133 |
|  | Co^2+^ | 0.756 | 0.876 | 0.874 | 0.229 |
| Cao et al. | Ca^2+^ | 0.595 | 0.792 | 0.789 | 0.125 |
|  | Mg^2+^ | 0.502 | 0.819 | 0.816 | 0.087 |
|  | Mn^2+^ | 0.765 | 0.798 | 0.798 | 0.160 |
|  | Zn^2+^ | 0.941 | 0.843 | 0.844 | 0.253 |
|  | Fe^3+^ | 0.879 | 0.727 | 0.729 | 0.158 |
|  | Fe^2+^ | 0.901 | 0.736 | 0.739 | 0.171 |
|  | Cu^2+^ | 0.917 | 0.829 | 0.830 | 0.246 |
|  | Na^+^ | 0.333 | 0.782 | 0.775 | 0.035 |
|  | K^+^ | 0.456 | 0.628 | 0.623 | 0.030 |
|  | Co^2+^ | 0.732 | 0.823 | 0.822 | 0.176 |
| Wang et al. | Ca^2+^ | 0.686 | 0.657 | 0.657 | 0.095 |
|  | Mg^2+^ | 0.695 | 0.770 | 0.769 | 0.114 |
|  | Mn^2+^ | 0.831 | 0.769 | 0.770 | 0.163 |
|  | Zn^2+^ | 0.921 | 0.897 | 0.898 | 0.310 |
|  | Fe^3+^ | 0.838 | 0.861 | 0.861 | 0.231 |
|  | Fe^2+^ | 0.926 | 0.872 | 0.873 | 0.275 |
|  | Cu^2+^ | 0.907 | 0.906 | 0.906 | 0.335 |
|  | Na^+^ | 0.642 | 0.654 | 0.654 | 0.078 |
|  | K^+^ | 0.688 | 0.713 | 0.712 | 0.152 |
|  | Co^2+^ | 0.738 | 0.827 | 0.826 | 0.180 |
| Hu et al. | Ca^2+^ | 0.600 | 0.793 | 0.790 | 0.127 |
|  | Mg^2+^ | 0.757 | 0.840 | 0.839 | 0.172 |
|  | Mn^2+^ | 0.768 | 0.802 | 0.801 | 0.162 |
|  | Zn^2+^ | 0.781 | 0.827 | 0.827 | 0.187 |
|  | Fe^3+^ | 0.909 | 0.835 | 0.836 | 0.230 |
|  | Fe^2+^ | 0.963 | 0.918 | 0.919 | 0.359 |
|  | Cu^2+^ | 0.741 | 0.768 | 0.767 | 0.152 |
|  | Na^+^ | 0.432 | 0.845 | 0.839 | 0.095 |
|  | K^+^ | 0.512 | 0.731 | 0.724 | 0.094 |
|  | Co^2+^ | 0.768 | 0.836 | 0.834 | 0.196 |
